# Theory of Mind and Its Elusive Structural Substrate

**DOI:** 10.3389/fnhum.2021.618630

**Published:** 2021-03-02

**Authors:** Fernando Lizcano-Cortés, Jalil Rasgado-Toledo, Averi Giudicessi, Magda Giordano

**Affiliations:** ^1^Laboratory of Neural Plasticity, Instituto de Neurobiología, Department of Behavioral and Cognitive Neurobiology, Universidad Nacional Autónoma de México, Querétaro, Mexico; ^2^Department of Psychology, Benemérita Universidad Autónoma de Puebla, Puebla, Mexico

**Keywords:** structural magnetic resonance imaging, voxel-based morphometry, cortical thickness, theory of mind, reproducibility

## Abstract

Is brain structure related to function? Can one predict the other? These are questions that are still waiting to be answered definitively. In this paper we seek to investigate these questions, in particular, we are interested in the relation between brain structure and theory of mind (ToM). ToM is defined as the ability to attribute mental states to others. Previous studies have observed correlations between performance on ToM tasks, and gray-matter size/volume in dorsomedial prefrontal cortex (dmPFC), temporoparietal junction (TPJ) and precuneus (PCu). Despite these findings, there are concerns about false positive results and replicability issues. In this study we used two different tasks to evaluate ToM, Reading the Mind in the Eyes Test (RMET), and the Short Story Task (SST). Performance in these tasks was correlated to brain anatomy measures including voxel-based morphometry (VBM) and cortical thickness (CT) analysis, from ninety-one neurotypical participants. High-resolution structural brain images were acquired, and whole-brain and region of interest (ROI) analyses were implemented. The analyses did not show statistically significant associations between ToM performance and brain structural measures after correction. Significant associations between performance on ToM tests and a widespread array of regions loosely associated with ToM were observed only for whole brain uncorrected analysis (*p* < 0.001). These results do not replicate a previous study with neurotypical participants. We tested two different ToM tests, two different softwares for VBM and CT, and we used two samples, one with 91 and a sub-sample with 69 participants. Neither of these conditions made a difference in the results obtained. Consequently, these results suggest that if the population is neurotypical and homogenous, it is unlikely that a reliable association between brain anatomy measures and ToM performance, as measured with these tasks, may be found.

## 1. Introduction

The theory of the mind (ToM), also known as mentalizing, is defined as the ability to attribute mental states to others (Premack and Woodruff, 1978; Frith and Frith, 2006) and to obtain knowledge about others' perspectives at a given moment or in a particular situation, including intentions, hopes, expectations, fantasies, desires, or beliefs. This ability is essential for successful navigation in social life (Leslie, 2000; Krawczyk, 2018). These mental states can be divided into two components, an affective one, which involves the understanding of emotions, feelings or affective states and a cognitive component that implies beliefs, thoughts or intentions (Henry et al., 2015). There are several psychometric instruments that have been used to measure ToM such as the Reading the Mind in the Eyes Test (RMET) for the affective component (Baron-Cohen et al., 2001), and the Short Story Task (SST) to evaluate the cognitive component (Dodell-Feder et al., 2013). Behavioral and clinical researchers have described this measurable and consistent cognitive ability, and impairments in this cognitive process for some disorders, which compromise social interactions (Chung et al., 2014). In a previous behavioral study we showed the concurrent validity of these two tests (Giordano et al., 2019) in our sample of Mexican participants, and found results similar to those published by Dodell-Feder et al. (2013).

Functional neuroimaging and structural connectivity studies have identified dorsal medial prefrontal cortex (dmPFC) and temporoparietal junction (TPJ) as the core regions of the neural substrate for ToM, extending to regions that include the precuneus (PCu), anterior temporal cortex, anterior cingulate and posterior cingulate (PostCing), medial prefrontal cortex (mPFC), orbitofrontal cortex (OFC), inferior frontal gyrus (IFG) and amygdala, to constitute an extended ToM neural network (Molenberghs et al., 2016; Wang and Olson, 2018). It's important to note, neuroimaging studies have found different results depending on the type of ToM task and presentation modality (Schurz et al., 2014).

Functional findings have led to questions about the structural correlate of ToM. This research suggested that cognitive functions may be related to the size/volume of a particular brain structure (Kanai and Rees, 2011; Qing and Gong, 2016). Previous studies using structural magnetic resonance imaging (sMRI) techniques, such as voxel based morphometry, have observed positive correlations between performance on RMET and gray-matter (GM). Sato et al. (2016) found correlations between ToM performance and volume of dorsomedial prefrontal cortex (dmPFC), inferior parietal lobule (i.e., TPJ) and PCu. However, Yin et al. (2018) found stable correlations only with left posterior superior temporal sulcus (TPJ in Sato et al., 2016). Additionally, GM in extended ToM regions involved in social cognition such as dmPFC and OFC, correlate with the size of social networks of individuals (Kwak et al., 2018).

Studies in social cognition and ToM have described training specific (short daily mental practices) increases in cortical thickness, in frontoinsular regions for socio-affective training, and in inferior frontal and lateral temporal cortex after socio-cognitive training (Valk et al., 2017). Others have found a negative correlation between cortical thickness and a measure of spontaneous ToM (STOMP) in mPFC and right IFG (Rice and Redcay, 2015), and left-hemispheric thinning in areas related to ToM, middle frontal gyrus (MFG), inferior parietal lobe (IPL), inferior temporal gyrus (IFG), and superior temporal gyrus (STG), and superior frontal gyrus (SFG) (Richter et al., 2015). These results suggest though there is a possible structural correlate of functionality for ToM, the existing literature demonstrates that there is still no consensus.

It has been argued that inter-individual variability in cognitive functions can be predicted from white and gray-matter anatomy (Kanai and Rees, 2011), however the issue of replicability has been questioned, especially in studies with small samples and neurotypical participants (Masouleh et al., 2019). Using data from hundreds of healthy individuals, Masouleh et al. (2019) found few statistically significant brain-behavior associations, and difficulty replicating those results in independent samples.

To address the relationship of cognitive processes with brain structure, and its replicability, in the present study we measured ToM abilities using two different tasks of ToM in a medium sized sample (*n* = 91) of neurotypical participants. Then, we acquired brain images using MRI in order to analyze the association between the RMET and SST-MSR (mental state reasoning) scores and gray-matter volume using two sMRI techniques, voxel-based morphometry and cortical thickness analysis, which have previously shown significant associations with ToM.

## 2. Materials and Methods

### 2.1. Subjects

Our sample consisted on ninety-one neurotypical native Mexican participants (52 females), aged 18–28 (median = 22), right-handed assessed by the Edinburgh Handedness Inventory (Oldfield, 1971), 12–21 years of education (median = 16), with no psychological distress (Cruz-Fuentes et al., 2005; González-Santos et al., 2007) (SCL-90 mean = 0.57, SD = 0.37), normal verbal comprehension measured by the Wechsler Adult Intelligence Scale (Wechsler, 2012) (mean = 108.41, SD = 10.93)(Supplementary Figure 8), and with no structural brain abnormalities observed by a visual inspection of all structural images. All participants answered the Short Story Task (SST), of which sixty-nine also answered the Reading the Mind in the Eyes Test (RMET) (40 females, median age = 22). All participants signed an informed consent form approved by the internal Committee on Ethics (47.H-RM), which also approved the experimental protocol, in compliance with the federal guidelines of the Mexican Health Department (http://www.diputados.gob.mx/LeyesBiblio/regley/Reg_LGS_MIS.pdf), which agree with international regulations. They were recruited through announcements in nearby universities, places of interest and by word of mouth.

### 2.2. ToM Tasks

RMET was used to measure the affective component of ToM (Baron-Cohen et al., 2001), while the SST was used to evaluate the cognitive component (Dodell-Feder et al., 2013). The RMET comprises 36 photographs depicting the region of the eyes. Subjects were asked to select from four mental state terms, presented in each corner or the screen, the term that best matched the mental state of the actor, as described previously in Giordano et al. (2019). Psychopy v.1.83.01 implemented on Windows 10 operating system, was used to present photographs and register the answer. In the SST, participants were asked to read a story written by Ernest Hemingway and to answer questions that assess explicit mental state reasoning (MSR) and comprehension (Dodell-Feder et al., 2013; Giordano et al., 2019). All 91 participants answered the SST, and a subsample of 69 participants answered both the SST and the RMET. Because not all of our participants responded to the RMET, we were able to segregate our participants into a large full sample of 91, and a subsample of 69 and compare the correlation results obtained for SST with VBM and CT.

### 2.3. Processing

**Figure 1:**
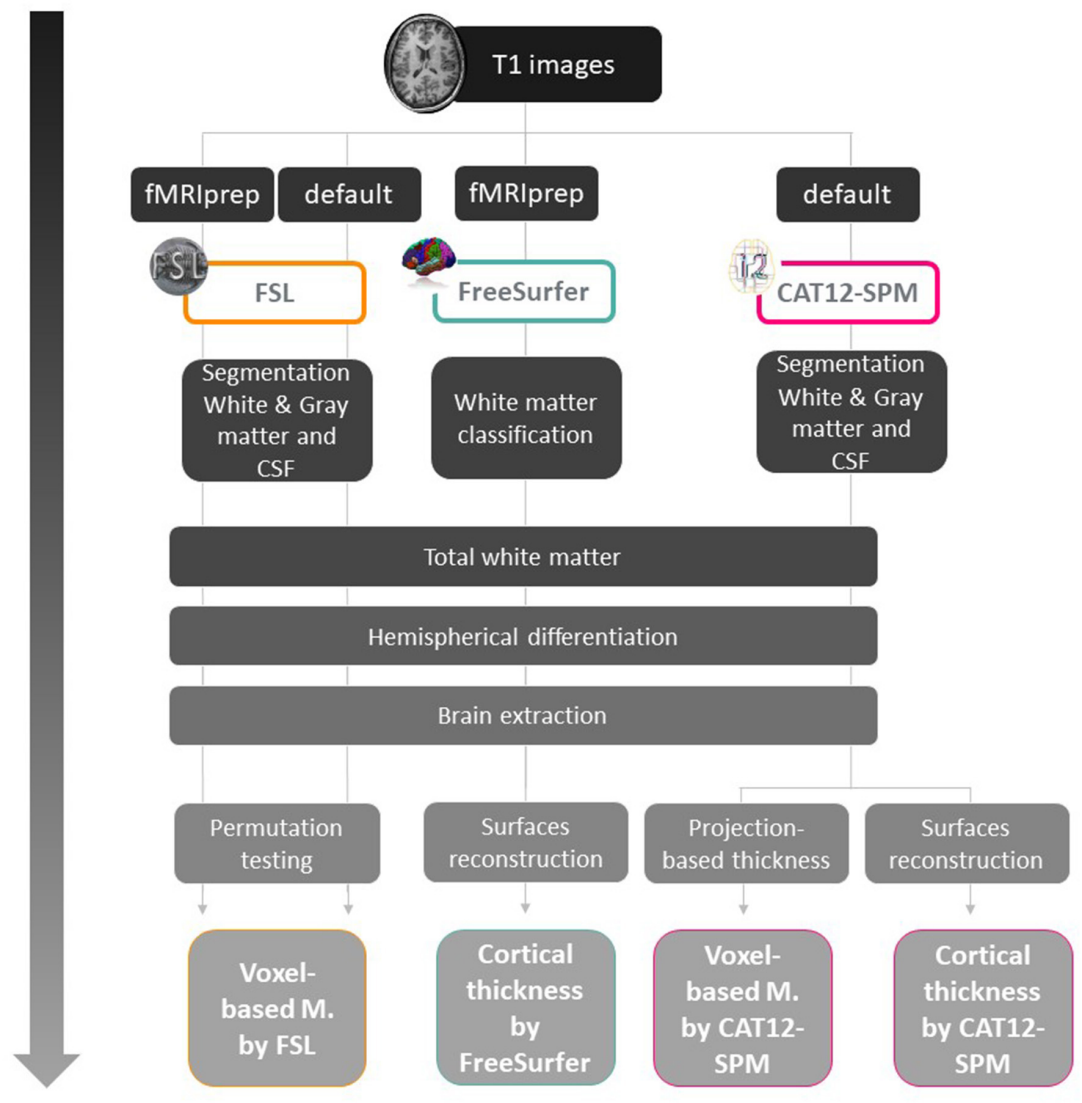
Workflow for cortical thickness and voxel-based analysis for each software. fMRIprep: functional magnetic resonance imaging data preprocessing pipeline (Esteban et al., 2019). This pipeline was used because in addition to the structural scans, task-related fMRI images were acquired for other studies.

The present study was part of a large project cohort that included several task-related functional MRI paradigms, and involved the scanning of different MRI-sequences including resting state images and high-resolution structural images for each participant. We preprocessed all MRI images obtained for all subjects using the standard steps for multimodal scanning in fMRIprep which includes steps for processing high-resolution structural images. However, for FreeSurfer and SPM12, preprocessing of structural MRI images followed a specific workflow for each type of analysis and software, as shown in Figure 1 and Supplementary Material.

Two types of analysis were implemented for VBM and CT (Figure 1), the first analysis was on the whole brain and the second was region of interest (ROI) analysis using masks and extracting values. In order to verify brain areas that have been described in ToM functional activation tasks, for ROI analysis, six maximum peaks were selected using Neurosynth meta-analysis data for the term “*mentalizing”* (Yarkoni et al., 2011), and three ROIs reported in a similar previous study (Sato et al., 2016), with a total of nine ROIs. For multiple comparisons, we implemented different corrections according to the recommendations for each software: TFCE for FSLVBM, FWE for CAT12 and FDR for FreeSurfer.

### 2.4. Voxel-Based Morphometry (VBM)

From the fMRIprep output we excluded brain extraction (BET), and segmented-registration steps, instead we used MNI152NLin2009cAsym standard space for registering GM images, permutation testing, and inference using threshold-free cluster enhancement (TFCE) for cluster-based correction. Different levels of smoothing (3, 5, 10 mm) were tested to verify differential results, finding similar effects for all of them. Results are presented with 10 mm smoothing. For VBM analysis two softwares were used: FSL-VBM processing was implemented with the default pipeline (https://fsl.fmrib.ox.ac.uk/fsl/fslwiki/FSLVBM) and CAT12-VBM toolbox was implemented to extract total intracranial volume (TIV) as the sum of the GM, WM, and CSF.

For the FSL-VBM tool, we tested two pre-processing methods finding similar results, thus for further analyses we used those obtained with the fMRIprep pipeline.

For ROI analysis we constructed anatomical spherical masks of 10 mm in FSL-VBM and 12 mm radius in CAT12, centered on coordinates, shown in Table 1. We extracted brain volume of ROI using fslstats function for FSL and marsbars for SPM toolbox for MATLAB, to compare differences between both softwares and to test the reliability of our results.

**Table 1:** Regions of interest extracted from Neurosynth using the term “mentalize” and from Sato et al. (2016), as well as their abbreviations and coordinates in Harvard-Oxford cortical atlas.

			**Coordinates**		
	**name**	**Abbr**	**X**	**Y**	**Z**
NeuroSynth	Left medial frontal cortex	mFC-L	−4	48	−18
	Right lateral occipital cortex	LOC-R	54	−66	42
	Left temporal lobe	TL-L	−50	6	−32
	Left precuneus	PCu-L1	−2	−54	40
	Left angular gyrus	AnG-L	−50	−56	40
	Right middle temporal gyrus	MTG-R	50	4	−32
Sato et al. (2016)	Left dorsomedial prefrontal cortex	dmPFC-L	−9	14	52
	Left inferior parietal lobule	IPL/TPJ-L	−51	−48	28
	Left precuneus	PCu-L2	−5	49	66

### 2.5. Cortical Thickness (CT)

For cortical thickness analysis, we used CAT and Freesurfer softwares. In Surface Based Morphometry (CAT12-SBM) we extracted surface parameters and ran statistical analysis with the same multiple regression matrix without TIV as covariate, as recommended in the software manual. For FreeSurfer analysis, cortical reconstruction was enabled in the fMRIprep pipeline which includes intensity bias field removal, neuroanatomical label for each voxel, constructing models of the cortical surface, and inter-subject registration to fsaverage5 standard space. In this case TIV was added as a no-interest covariate in the design matrix. Volumes were visually inspected for misclassifications and manual error correction was performed following standard procedures (http://surfer.nmr.mgh.harvard.edu/). For both softwares, cortical thickness was calculated as the distance between white and pial surface with a smoothing of 15 mm FWHM Gaussian kernel on each hemisphere.

To compare the results for CT between softwares, we extracted 74 ROIs for each hemisphere according to Destrieux et al. (2010) atlas provided for FreeSurfer and CAT12-SBM softwares. As an additional analysis, we extracted brain cortical thickness of six ROIs (average of both hemispheres), previously associated with ToM, to compare differences between both softwares, the correlation with ToM scores, and the reliability of our results. The ROI included IFG, MFG, OFC, IPL/TPJ, PCu, and middle temporal gyrus (MTG), which are described in Table 1.

The association between results of VBM and CT analyses and ToM tasks scores was evaluated with multiple regression using test scores as effect-of-interest independent variables, and age, sex, General Ability Index (WAIS-IV), and TIV were added as no-interest covariates for each of two kind of software used. Voxels were considered statistically significant if they passed the threshold (*p* < 0.05) using family-wise error in CAT12 or false discovery rate in FreeSurfer for multiple comparisons corrections. Uncorrected threshold was set at *p* < 0.001.

### 2.6. Power Analysis

With regard to the power of our study, we calculated the effect size estimate according to Lakens (2013) for within-subjects designs. Cohen's dz from the smallest t value reported by Sato et al. (2016), which was *t* = 3.08, yielded a medium effect size (d = 0.43) that would require 44 participants for a *p* < 0.05 and power of 0.80 (calculated with R v. 3.6, library pwr).

## 3. Results

### 3.1. Behavioral Results

Means and standard deviations were calculated for SST subtests and RMET scores. They are listed in Table 2.

**Table 2:** Means, standard deviations and ranges of theory of mind measures are shown for the sub-sample of 69 volunteers for RMET (range = 0–36), and for SST (ranges: SI = 0–1; MSR = 0–16; C = 0–10; SST = 0–27) and for the sample of 91 volunteers for SST.

		**Mean**	**SD**	**Range**
RMET (*n* = 69)		26.06	3.36	17–32
SST (*n* = 69)	Spontaneous inference	0.16	0.37	0–1
	Comprehension	7.28	1.98	3–10
	Mental state reasoning	6.75	2.25	2–12
	Short story total	14.19	3.44	5–22
SST (*n* = 91)	Spontaneous inference	0.4	0.35	0–1
	Comprehension	7.14	1.89	3–10
	Mental state reasoning	7.82	2.85	2–14
	Short story total	15.11	3.66	5–23

### 3.2. Neuroanatomical Results

**Figure 2:**
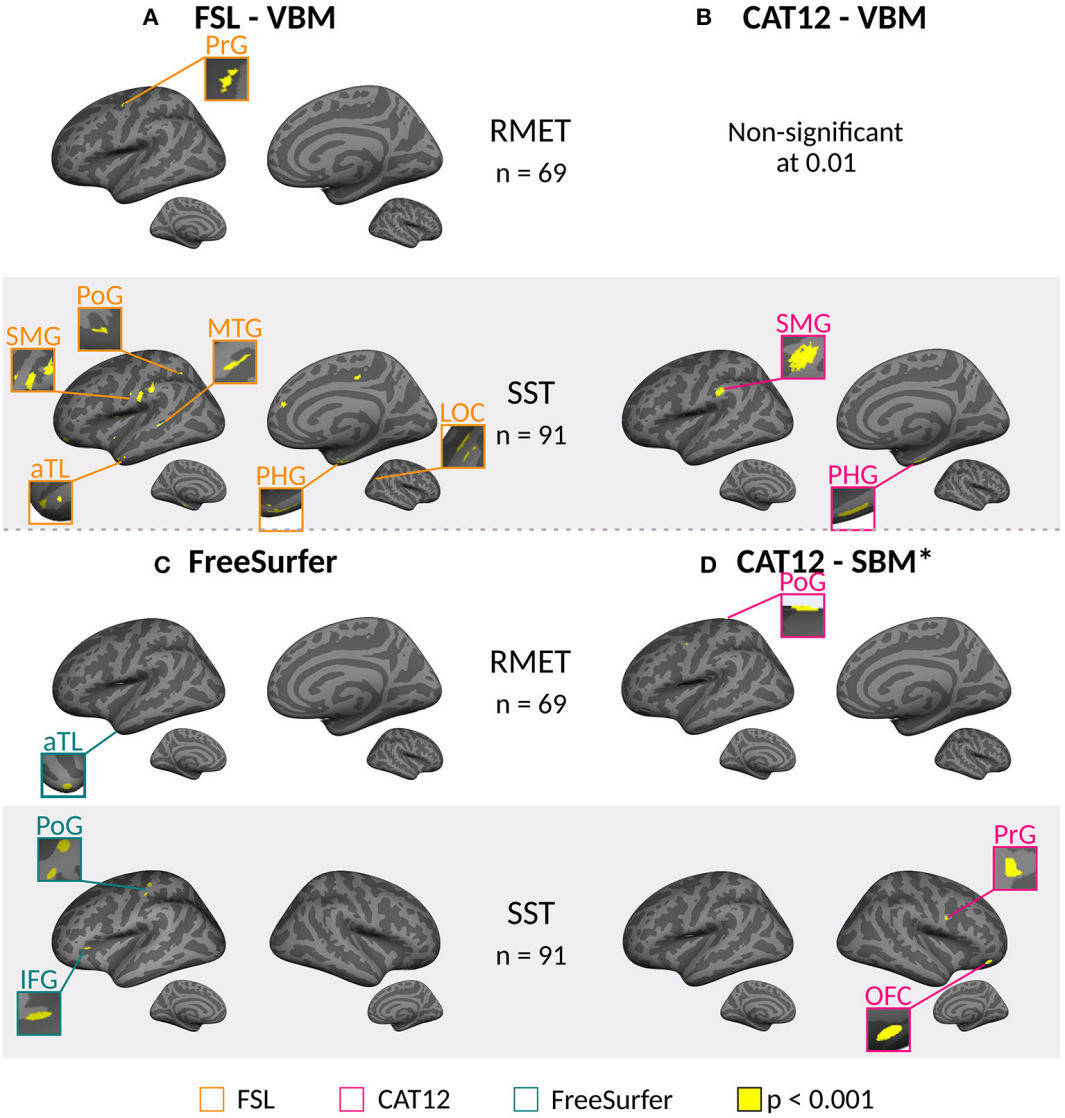
Uncorrected significant clusters in brain areas correlated with mental state reasoning scores for the Short Story Task (SST-MSR), and Reading Mind in the Eyes Test (RMET) scores for voxel based morphometry (VBM) and cortical thickness analysis (CT). Cortical inflated reconstruction was performed with FreeSurfer using a MNI152 template (https://surfer.nmr.mgh.harvard.edu/fswiki/FsTutorial/Visualization). **(A)** Clusters found using FSL-VBM toolbox. **(B)** Significant clusters were found using CAT12-VBM MATLAB toolbox only for SST. No significant results were found with RMET values. **(C)** Clusters found using FreeSurfer for CT. **(D)** Clusters found using CAT12 Surface Based Morphometry MATLAB toolbox for CT. aTL, anterior Temporal Lobe; IFG, inferior frontal gyrus; LOC, lateral occipital cortex; MTG, middle temporal gyrus; OFC, orbitofrontal cortex; PHG, parahippocampal gyrus; PoG, postcentral gyrus; PrG, precentral gyrus; SMG, supramarginal gyrus. *Threshold set at *p* < 0.001. Brain areas clusters are listed in Supplementary Tables 1–10.

The whole brain analyses did not show statistically significant associations between performance on the ToM tests and structural measures after correction either for VBM or CT. This was the case for all preprocessing conditions and all softwares, and for the full sample of 91 participants, and the subsample of 69 participants. Uncorrected analyses (*p* < 0.001) showed significant associations with several regions for whole brain analysis, in both ToM tasks, described below (Figure 2). Analyses for the ROI masks did not show significant association with ToM scores even without correction.

#### 3.2.1. Voxel-Based Morphometry (VBM)

For the **whole brain** analysis, FSL-VBM and CAT12-VBM showed uncorrected correlations in areas loosely associated with ToM in both hemispheres.

These uncorrected results using a threshold of *p* < 0.001, showed that gray-matter volume measured by FSL-VBM in the left precentral gyrus (PrG) correlated with scores for the RMET for the subsample of 69 participants, (Figure 2A, Supplementary Table 1). Gray-matter volume in the right anterior temporal lobe (aTL), lateral occipital cortex (LOC), parahippocampal gyrus (PHG), and in the left frontal pole, PHG, postcentral gyrus (PoG), MTG, and supramarginal gyrus (SMG) correlated with SST-MSR scores of 91 participants (Figure 2A, Supplementary Table 2). Considering only the subsample of 69 participants that also answered the RMET, the results were similar, gray-matter volume in the right aTL, PHG, and MTG, and the left PoG, correlated with SST-MSR scores (Supplementary Table 3).

Using CAT12-VBM, there were no uncorrected correlations that passed the threshold (*p* < 0.001) between gray-matter volume and scores on either ToM task, for the subsample of 69 participants. However, uncorrected correlations between gray-matter volume in the left SMG and right PHG were found with SST-MSR scores (91 participants) (Figure 2B, Supplementary Table 4).

With regard to the **ROI analysis**, there were no significant correlations between gray-matter volumes, measured with both softwares, and ToM scores in either test, RMET or SST-MSR, with either the full sample or the subsample (Supplementary Figures 1, 2).

With regard to the comparison between softwares, the mean volume of the nine ROI were significantly different for the 91 participants (Supplementary Figure 3), FSL-VBM (mean = 0.518, SD = 0.13) and CAT12-VBM (mean = 0.387, SD = 0.09) with higher values for FSL [*F*_(8, 1)_ = 53.25329, effect size (*ges*) = 0.266, *p* < 0.001].

For the nine ROI explored, for 69 subjects, there was a significant negative correlation between softwares for mean volume in the PCu-L1 peak [*r*(67) = −0.383, *p* < 0.001] (Supplementary Figure 1). In contrast, only PCu-L2 [*r*(89) = 0.56, *p* < 0.001] and TL-L VBM [*r*(89) = 0.52, *p* < 0.001] values were significantly correlated between softwares when the full sample was used (Supplementary Figure 2).

#### 3.2.2. Cortical Thickness (CT)

For the **whole brain** analysis, FreeSurfer showed uncorrected correlation between CT in the right aTL and RMET scores for the subsample (Figure 2C, Supplementary Table 5). For the full sample (91 participants), CT in the left PoG and IFG, correlated with the scores for SST-MSR (Figure 2C, Supplementary Table 6); for the subsample (69 participants) CT in the right PrG and Cuneus showed correlation with these scores (Supplementary Table 7).

The results obtained with CAT12-SBM showed correlations between CT in the left PoG and RMET scores (Figure 2D, Supplementary Table 8), between CT in the right OFC and PrG and SST-MSR scores with all 91 participants (Figure 2D, Supplementary Table 9), and between CT in the right MFG and SST-MSR scores with 69 participants (Supplementary Table 10).

For the **ROI analysis**, considering the subsample of 69 participants, CT in MTG as measured by Freesurfer was significantly (*p* < 0.001) but negatively correlated with RMET scores [*r*(67) = −0.230] (Supplementary Figure 4). Considering all 91 participants, significant (*p* < 0.001) but negative correlations were found between CT in PCu and SST-MSR scores with both softwares [*FS*:*r*_(89)_ = −0.162;*CAT*:*r*_(89)_ = −0.118], and between CT in IPL/TPJ measured with FreeSurfer and SST-MSR scores [*r*(89) = −0.101] (Supplementary Figure 5). No correlations between CT and SST-MSR scores were found with the subsample.

With regard to the comparison between softwares, density of all 74 ROI for each hemisphere according to Destrieux et al. (2010) are presented in Supplementary Figure 6. Mean cortical thickness measured with CAT12-SBM was 2.89 (*SD* = 0.46), for FreeSurfer it was 2.54 (*SD* = 0.36), and they were significantly different [*F*_(73, 1)_ = 242.3031, effect size (*ges*) = 0.155, *p* < 0.001]. For illustration purposes, CT values for 41 representative ROI are shown in Supplementary Figure 7 (91 participants).

Significant (*p* < 0.001) positive correlations between Free Surfer and CAT12-SBM for CT were found for IFG [*r*(67) = 0.756], MFG [*r*(67) = 0.665], OFC [*r*(67) = 0.674], IPL/TPJ [*r*(67) = 0.806], and PCu [*r*(67) = 0.782] for the subsample of 69 participants (Supplementary Figure 4). Whereas, significant positive correlations were found between softwares for CT in IPL/TPJ [*r*(89) = 0.770] and PCu [*r*(89) = 0.766], for the full sample of 91 participants (Supplementary Figure 5).

## 4. Discussion

The relation between brain structure and function is relevant for understanding the neural basis of cognition (Kanai and Rees, 2011). Neuroimaging techniques have provided the means to obtain detailed morphometric information for characterizing individual differences in brain anatomy, and also evidence of a relationship between brain structure and humans skills and traits (Kanai and Rees, 2011; Masouleh et al., 2019). However, the replicability of significant associations between structure and function is low, and influenced by methodological and conceptual factors (Masouleh et al., 2019).

Underscoring the issue of low replicability of previous findings, the results of the present study do not replicate those of a previous one (Sato et al., 2016) that found an association between brain anatomy and Theory of Mind measures (*n* = 51), specifically, between VBM and RMET. It must be noted, however, that Sato et al. (2016) did not find significant correlations when using a whole-brain analysis. Our results agree with those by Rice and Redcay (2015) who found no association between ROI thickness and RMET performance (*n* = 38). The differences in results cannot be attributed to differences in characteristics of the sample in each study.

With regard to our sample size, since this was a replication study, we calculated the effect size estimate from the values reported by Sato et al. (2016), yielding a medium effect size that would require 44 participants for a *p* < 0.05 and power of 0.80, therefore the size of the subsample (*n* = 69) and full sample (*n* = 91) were adequate. Also, it has been observed that when studies are underpowered, subsequent replication studies tend to find smaller effects or even contradict findings from the initial study (Button et al., 2013, for an in-depth discussion of these issues).

We corroborated these results using two different softwares to estimate local gray-matter volume, the VBM tool in FSL v.5.0.6 and the Computational Anatomy Toolbox (CAT12-VBM) tool r1450 in MATLAB R2018b. We also tested two different pre-processing methods for the FSL-VBM tool, finding similar results, thus for further analyses we used the fMRIprep pipeline. Results of the whole brain analysis were the same in all cases, no significant associations between brain morphometry and RMET scores after correction using family-wise error or false discovery rate for multiple comparisons. Similar corrections were used by Sato et al. (2016), and that in their study, the whole brain analysis yielded similar results to ours, no association with RMET scores. Significant associations were only found when an ROI approach was used for the dmPFC, inferior parietal lobule, and precuneus in the left hemisphere. In the present study, the ROI approach did not yield significant association between VBM and RMET scores.

In addition to the RMET that explores the affective component of ToM, we used the SST, a ToM task that explores the cognitive component, and that we had previously found to correlate with the RMET in a sample of 118 Mexican participants (Giordano et al., 2019). In the present study, we used the scores of 91 participants in this task, and similarly to what we found with RMET, there was no association between VBM and SST-MSR scores on the whole brain analysis or ROI approach.

We evaluated another measure of brain anatomy, cortical thickness. This varies between cortical areas and may reflect differences in cell types or neuron densities (Kanai and Rees, 2011). Two different softwares were also used, CAT12-SBM and FreeSurfer v.5.3. Results were the same as with VBM, no significant association between cortical thickness and RMET (69 participants) or SST-MSR (full sample of 91 participants or subsample of 69 participants), using either the whole brain analysis or the ROI approach.

We decided to explore the results of the uncorrected whole brain and ROI analyses, and the association with ToM scores, for the whole sample of 91 participants and the subsample of 69. The purpose was to compare between softwares, anatomical measures and sample sizes using a less conservative approach. For the whole brain analyses using FSL-VBM, there was no spatial overlap between brain areas associated with RMET and SST-MSR. For SST-MSR, there was partial spatial overlap for both sample sizes, the full sample showed more brain areas associated with the ToM scores. These brain regions included areas that previously associated with ToM such as the anterior temporal lobe, supramarginal gyrus, and cingulate cortex (Molenberghs et al., 2016; Wang and Olson, 2018). Uncorrected associations between CAT12-VBM, and SST-MSR scores were found in two of the same brain areas, supramarginal gyrus and parahippocampal gyrus, that showed an association using FSL-VBM, for the full sample only. ROI analyses did not show correlations with ToM scores.

In contrast to VBM, cortical thickness on the whole brain uncorrected analyses showed no spatial overlap between softwares. Cortical thickness measured with FreeSurfer in the anterior temporal lobe was associated with RMET scores, while cortical thickness in the right precentral gyrus and cuneus, and left post central gyrus and inferior frontal gyrus was associated with SST-MSR scores for the subsample and the full sample, respectively. With CAT12-SBM, cortical thickness in the left post central gyrus was associated with RMET scores. While cortical thickness in the right medial frontal gyrus, for the subsample, and the right orbitofrontal cortex and precentral gyrus, for the full sample, was associated with SST-MSR scores. The ROI analyses showed negative correlations between RMET scores and middle temporal gyrus measured with Freesurfer in the subsample. Also, negative correlations were found between SST-MSR scores and inferior parietal lobe and precuneus measured with FreeSurfer, and precuneus measured with CAT-12, considering the full sample.

In addition to evaluating the association between anatomical measures and ToM, we evaluated the similarity between softwares for the ROI analyses. Our results showed significant differences between softwares in the mean volume of nine ROI, considering 91 participants, with FSL-VBM showing greater values than CAT12-VBM, similar to a previous study (Rajagopalan et al., 2014). With regard to the correlation between softwares, only two areas out of nine, left precuneus and left temporal lobe, were significantly and positively correlated between FSL-VBM and CAT12-VBM considering the full sample. In contrast, with the subsample, significant negative correlation between softwares was found for left precuneus.

In terms of cortical thickness, mean cortical thickness also showed differences between CAT12-SBM and FreeSurfer in the 74 regions from the aparc.a2009s atlas (Destrieux et al., 2010). Correlations between softwares varied according to sample size. While there was a significant positive correlation between softwares for inferior parietal lobe, and precuneus for the full sample. For the subsample, significant positive correlations were found for inferior frontal gyrus, medial frontal gyrus, orbitofrontal cortex, inferior parietal lobe and precuneus. We did not find significant correlations in VBM measures between softwares.

Different computational programs can provide different estimations of volume/size based on the methods used by the program to obtain the measures (Rajagopalan et al., 2014; Righart et al., 2017; Seiger et al., 2018; Guo et al., 2019). Despite the fact that Freesurfer is not considered the “gold standard,” its measurements are generally taken as accurate and robust (Rajagopalan et al., 2014). The Computational Anatomy Toolbox (CAT12) is a relatively new volume based approach that uses projection-based-thickness where a central surface, which has better properties than white-matter, is generated at 50% distance between gray-matter and cerebral spinal fluid and includes the estimation of the CT and the central surface of both hemispheres (Seiger et al., 2018).

Our results are consistent with previous studies that found CAT12 analysis showing generally greater cortical thickness in ROI (Rajagopalan et al., 2014; Seiger et al., 2018), and significant correlation between FreeSurfer and CAT12 results, in agreement with a previous study (Seiger et al., 2018). but this was dependent on the sample size. Given that both softwares provide similar robustness and have excellent test-retest scores (Seiger et al., 2018; Guo et al., 2019), it is considered that both approaches provide similar and powerful estimates of surface based assessments.

The main limitation of this study is the sample size, although we used a subsample to test the consistency of our results, this was not done randomly. A larger sample would have allowed replication with independent matched subsamples of different sizes. Another limitation was the tasks used for ToM, although the RMET is one of the most used tests all over the world, it has a relatively low internal consistency, and there is the possibility that it may be measuring more than one factor (Giordano et al., 2019). The SST is a relatively new test (Dodell-Feder et al., 2013) that uses naturalistic narrative stimuli to assess the ability to attribute emotional states, beliefs and intentions to the characters in the story. Also, in contrast to our previous study, with 118 volunteers (Giordano et al., 2019), the scores in these tasks did not show a significant correlation in the sample of 69 volunteers reported in this study. Since ToM is a multidimensional construct (Turner and Felisberti, 2017) each task is likely to measure a different aspect of it, and as has been suggested by others, psychological measures have not been developed to identify specific localized brain functions (Masouleh et al., 2019). In spite of these results, it must be noted that with some exceptions (i.e., PrG, PoG), the areas found to be associated with ToM scores are those that have been previously associated with this ability. Thus, it is possible that a study using a larger, and more diverse sample with a greater variety of ToM tests may be able to find a significant association.

In conclusion, this study found no significant corrected associations between brain anatomy and ToM scores. Uncorrected analyses between gray-matter volume, cortical thickness and ToM scores showed very little spatial overlap. Evaluation of ROI gray-matter volume and cortical thickness showed different results depending on the software, and no consistent association with ToM scores. Findings using the full sample were not always consistent with those found using the subsample. Although our study was of limited scope, our results agree in general with the conclusions by Masouleh et al. (2019) after their empirical investigation assessing the replicability of structural brain behavior associations. Briefly, that finding an association between performance at standard psychological tests, and brain morphology among healthy individuals is relatively unlikely, and that answering the question about brain behavior associations, requires substantially large samples. These authors encourage the reporting of null findings, such as ours, to contribute to shape a more objective picture of this association.

## Data Availability Statement

The datasets presented in this study can be found in online repositories. The names of the repository/repositories and accession number(s) can be found at: https://openneuro.org/datasets/ds003481/versions/1.0.2.

## Ethics Statement

The studies involving human participants were reviewed and approved by Comité de Ética en Investigación, Instituto de Neurobiología, UNAM. The patients/participants provided their written informed consent to participate in this study.

## Author Contributions

MG conceived the experiments. MG, FL-C, and JR-T designed the experiments. FL-C, JR-T, and AG curated the data and wrote the manuscript. FL-C and JR-T ran the experiments and analyzed the data. MG supervised the entire process. All authors contributed to interpretation of data, revised the manuscript, and have read and approved the final manuscript.

## Conflict of Interest

The authors declare that the research was conducted in the absence of any commercial or financial relationships that could be construed as a potential conflict of interest.
