# Supplementary material for: Theory of Mind and Its Elusive Structural Substrate
Source: Front Hum Neurosci. 2021 Mar 2;15:618630. doi: 10.3389/fnhum.2021.618630 (PMC7982864; doi:10.3389/fnhum.2021.618630)
Supplement: Supplementary file 1 [file Data_Sheet_1.pdf]

Supplementary Material

1 SUPPLEMENTARY METHOD

1.1 MRI data acquisition

The present study was part of a large project cohort with several MRI functional tasks related with Pragmatic Language processing, and involved the scanning of different MRI-sequences including resting state images and high-resolution structural images for each participant.

All brain MRI data were acquired in a 3.0 T GE Discovery-MR750 in high-resolution structural 3D-T1-weighted images, using a 32-channel head coil, by a spoiled gradient-recalled (SPGR) sequence with the following parameters: 1 mm^3 isotropic voxels; Repetition Time = 2.3 ms (TR); Echo Time = 3 ms (TE), flip angle = 12.0^o; in-plane acquisition matrix = 256 x 256 mm, covering the whole brain.

1.2 Data analysis

1.2.1 Preprocessing

Image and statistical analyses for Voxel Based Morphometry (VBM) were performed using the VBM tool in FSL v.5.0.6 (RRID:SCR_002823, https://fsl.fmrib.ox.ac.uk/fsl/fslwiki/FSLM; Smith et al. (2004)) and Computational Anatomy Toolbox (CAT12-VBM) tool r1450 (http://www.neuro.uni-jena.de/cat/) within SPM12 r7758 (RRID:SCR_013273, http://www.fil.ion.ucl.ac.uk/spm/software/spm12/) in MATLAB R2018b (MathWorks Inc., Natick, MA USA). Analyses for cortical thickness were done with CAT12-SBM and FreeSurfer v.5.3 (RRID:SCR_001847, http://surfer.nmr.mgh.harvard.edu/; Dale et al. (1999)).

We preprocessed all MRI images obtained for the large cohort using the standard steps for multimodal scanning in fMRIprep which included steps for processing high-resolution structural images. However, FreeSurfer and SPM12 preprocessing followed a different workflow for each type of analysis, as shown in Figure 1. fMRIprep v1.1.4 (RRID:SCR_016216) (Esteban et al., 2019) preprocessing was performed with the default steps when using FSL-VBM and FreeSurfer. These steps included T1-weighted volume correction for intensity non-uniformity, skull-stripped, nonlinear asymmetrical template (version 2009c) using brain-extracted T1-weighted volume, recon-all for brain surfaces reconstruction and tissue segmentation of cerebrospinal fluid (CSF), white-matter (WM) and grey-matter (GM). For further details on the pipeline please refer to: https://fmriprep.readthedocs.io/. Additionally, to test the reliability of non-significant results, and to evaluate the potential influence of preprocessing steps on the results of the study, we performed a default preprocessing of the same steps now using the FSL-VBM default pipeline, showing similar results with larger clusters but with non-corrected significant results as well.

For CAT12, preprocessing was performed using the default settings. T1-weighted scans were normalized using a non-linear registration, corrected for bias field inhomogeneities and segmented into grey, white-matter and cerebrospinal fluid using an adaptive maximum a posteriori approach (AMAP; Rajapakse et al. (1997), then the Diffeomorphic Anatomical Registration Through Exponentiated Lie algebra algorithm (DARTEL; Ashburner (2007)) was used to normalize segmented scans into a standard stereotactic space MNI. The normalized modulated images of both tissues were resampled to a resolution of 1.5 mm x 1.5 mm x 1.5 mm and for VBM we smoothed using a 12 mm full-width at half-maximum isotropic Gaussian kernel (FWHM) to compensate for anatomical variability and 15 mm for cortical thickness.

REFERENCES

Ashburner, J. (2007). A fast diffeomorphic image registration algorithm. *NeuroImage* 38, 95–113. doi:10.1016/j.neuroimage.2007.07.007

Dale, A. M., Fischl, B., and Sereno, M. I. (1999). Cortical Surface-Based Analysis. *NeuroImage* 9, 179–194. doi:10.1006/nimg.1998.0395

Destrieux, C., Fischl, B., Dale, A., and Halgren, E. (2010). Automatic parcellation of human cortical gyri and sulci using standard anatomical nomenclature. *NeuroImage* 53, 1 – 15. doi:https://doi.org/10.1016/j.neuroimage.2010.06.010

Esteban, O., Markiewicz, C. J., Blair, R. W., Moodie, C. A., Isik, A. I., Erramuzpe, A., et al. (2019). fMRIPrep: a robust preprocessing pipeline for functional MRI. *Nature Methods* 16, 111–116. doi:10.1038/s41592-018-0235-4

Rajapakse, J. C., Giedd, J. N., and Rapoport, J. L. (1997). Statistical approach to segmentation of single-channel cerebral mr images. *IEEE Transactions on Medical Imaging* 16, 176–186. doi:10.1109/42.563663

Smith, S. M., Jenkinson, M., Woolrich, M. W., Beckmann, C. F., Behrens, T. E., Johansen-Berg, H., et al. (2004). Advances in functional and structural MR image analysis and implementation as FSL. *NeuroImage* 23, 208–219. doi:10.1016/j.neuroimage.2004.07.051

2 ABBREVIATIONS

Abbr = Name

IFG = Inferior Frontal Gyrus

MFG = Middle Frontal Gyrus

OFC = Orbitofrontal Cortex

IPL = Inferior Parietal Lobe

PCu = Precuneus

MTG = Middle Temporal Gyrus

dmPFC = Dorsomedial prefrontal cortex

TPJ = Temporoparietal junction

aTL = Anterior Temporal Lobe

LOC = Lateral Occipital Cortex

PHG = Parahippocampal Gyrus

PoG = Postcentral Gyrus

PrG = Precentral Gyrus

SMG = Supramarginal Gyrus

AnG = Angular Gyrus

mFC = Medial Frontal Cortex

PCu-L = Left Precuneus

3 SUPPLEMENTARY TABLES

Table S1. Results of whole brain analysis showing uncorrected values for the association between FSL-VBM in these brain regions and RMET in 69 participants. Cluster size threshold >20 voxels.

Voxels	Z	p-value	X	Y	Z	H	Atlas Harvard-Oxford
24	3.64	0.001	-38	-6	44	L	Precentral Gyrus

Table S2. Results of whole brain analysis showing uncorrected values for the association between FSL-VBM in these brain regions and SST in 91 participants. Cluster size threshold >20 voxels. WM = White-matter

Voxels	Z	p-value	X	Y	Z	H	Atlas Harvard-Oxford
259	3.85	0.001	36	4	-42	R	Anterior temporal lobe
110	4.37	0.001	-28	48	-12	L	Frontal Pole
57	3.61	0.001	-26	-26	-24	L	Parahippocampal Gyrus
47	4.21	0.001	-56	-16	28	L	Postcentral Gyrus
32	3.7	0.001	38	-76	22	R	Lateral Occipital Cortex
32	3.55	0.001	-56	-42	2	L	Middle Temporal Gyrus
29	3.94	0.001	-58	-30	32	L	Supramarginal Gyrus
27	3.74	0.001	28	-6	-32	R	Parahippocampal Gyrus
22	3.56	0.001	18	-24	38	R	Cingulate Gyrus (WM)

Table S3. Results of whole brain analysis showing uncorrected values for the association between FSL-VBM in these brain regions and SST in 69 participants. Cluster size threshold >20 voxels. WM = White-matter

Voxels	Z	p-value	X	Y	Z	H	Atlas Harvard-Oxford
87	4.13	0.001	24	-78	12	R	Lateral Occipital Cortex (WM)
73	3.47	0.001	38	-74	24	R	Lateral Occipital Cortex (WM)
50	4.2	0.001	28	-10	-32	R	Parahippocampal Gyrus
46	4.04	0.001	-56	-16	28	L	Postcentral Gyrus
35	3.43	0.001	40	14	-38	R	Anterior temporal lobe
21	4.21	0.001	68	-20	-26	R	Middle Temporal Gyrus
29	3.94	0.001	-58	-30	32	L	Supramarginal Gyrus
27	3.74	0.001	28	-6	-32	R	Parahippocampal Gyrus
22	3.56	0.001	18	-24	38	R	Cingulate Gyrus (WM)

Table S4. Results of whole brain analysis showing uncorrected values for the association between CAT12-VBM in these brain regions and SST in 91 participants. Cluster size threshold >20 voxels. WM = White-matter

Voxels	Z	p-value	X	Y	Z	H	Atlas Harvard-Oxford
503	3.77	0.0001	-68	-28	32	L	Supramarginal gyrus
464	3.67	0.0001	26	-15	-36	R	Parahippocampal gyrus

Table S5. Results of whole brain analysis showing uncorrected values for FreeSurfer in these brain regions and RMET in 69 participants. Cluster size threshold >20 voxels.

Voxels	Z	p-value	X	Y	Z	H	Atlas Harvard-Oxford
227	3.17	0.001	37	18	-46	R	Anterior temporal lobe

Table S6. Results of whole brain analysis showing uncorrected values for FreeSurfer in these brain regions and SST in 91 participants. Cluster size threshold >20 voxels.

Voxels	Z	p-value	X	Y	Z	H	Atlas Harvard-Oxford
166	3.84	0.001	-41	-31	67	L	Postcentral Gyrus
84	3.37	0.001	-37	33	1	L	Inferior Frontal Gyrus
83	3.29	0.001	-51	-22	59	L	Postcentral Gyrus
26	3.17	0.001	-19	-31	72	L	Postcentral Gyrus

Table S7. Results of whole brain analysis showing uncorrected values for FreeSurfer in these brain regions and SST in 69 participants. Cluster size threshold >20 voxels.

Voxels	Z	p-value	X	Y	Z	H	Atlas Harvard-Oxford
136	3.04	0.001	32	-21	44	R	Precentral Gyrus
128	3.17	0.001	6	-85	24	R	Cuneus

Table S8. Results of whole brain analysis showing uncorrected values for CAT12-SBM in these brain regions and RMET in 69 participants.

Voxels	Z	p-value	X	Y	Z	H	Atlas Harvard-Oxford
162	2.65	0.004	-11	-34	74	L	Postcentral gyrus

Table S9. Results of whole brain analysis showing uncorrected values for CAT12-SBM in these brain regions and SST in 91 participants. Cluster size threshold >20 voxels.

Voxels	Z	p-value	X	Y	Z	H	Atlas Harvard-Oxford
185	2.71	0.003	30	36	-8	R	Orbitofrontal gyrus
59	2.53	0.006	53	9	30	R	Precentral gyrus

Table S10. Results of whole brain analysis showing uncorrected values for CAT12-SBM in these brain regions and SST in 69 participants. Cluster size threshold >20 voxels.

Voxels	Z	p-value	X	Y	Z	H	Atlas Harvard-Oxford
78	3.4	0.0001	6	33	-17	R	Medial Frontal gyrus

4 SUPPLEMENTARY FIGURES

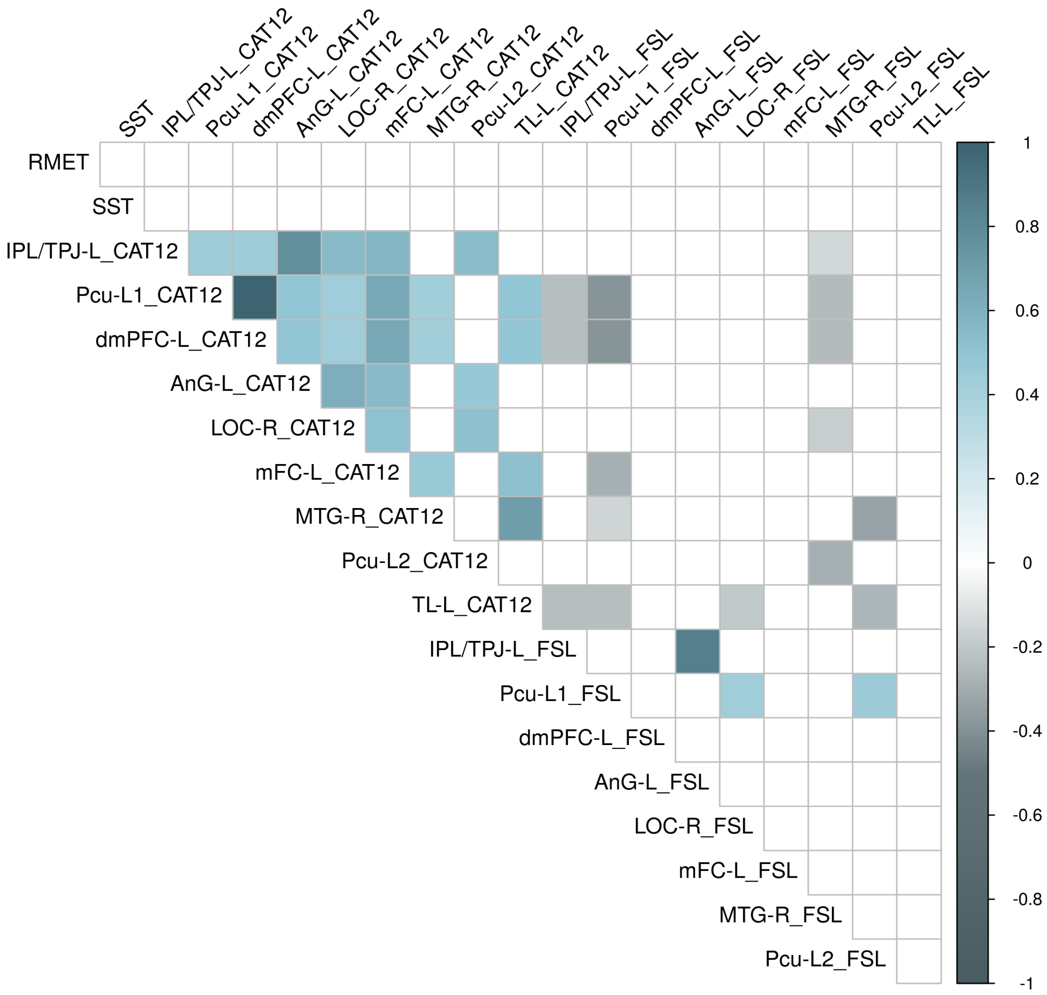

Figure S1. Correlations between extracted volumes (mm^3) of ROI using both softwares (FSL and CAT12), and Theory of Mind scores (RMET and SST-MSR) for the subsample (69 participants). Blank spaces means non-significant results ($p > 0.001$). Abbreviations: AnG-L: left Angular gyrus, dmPFC-L: left dorsomedial Prefrontal Cortex, LOC-R: right Lateral Occipital Cortex; mFC-L: left Medial Frontal Cortex, MTG-R: right Middle Temporal Gyrus, PCu-L1: left Precuneus (Yarkoni et al., 2011), PCu-L2: left Precuneus (Sato et al., 2016), TL-L: left Temporal Lobe, IPL/TPJ-L: left Inferior Parietal Lobule/Temporoparietal junction, CAT12: Computational Anatomy Toolbox.

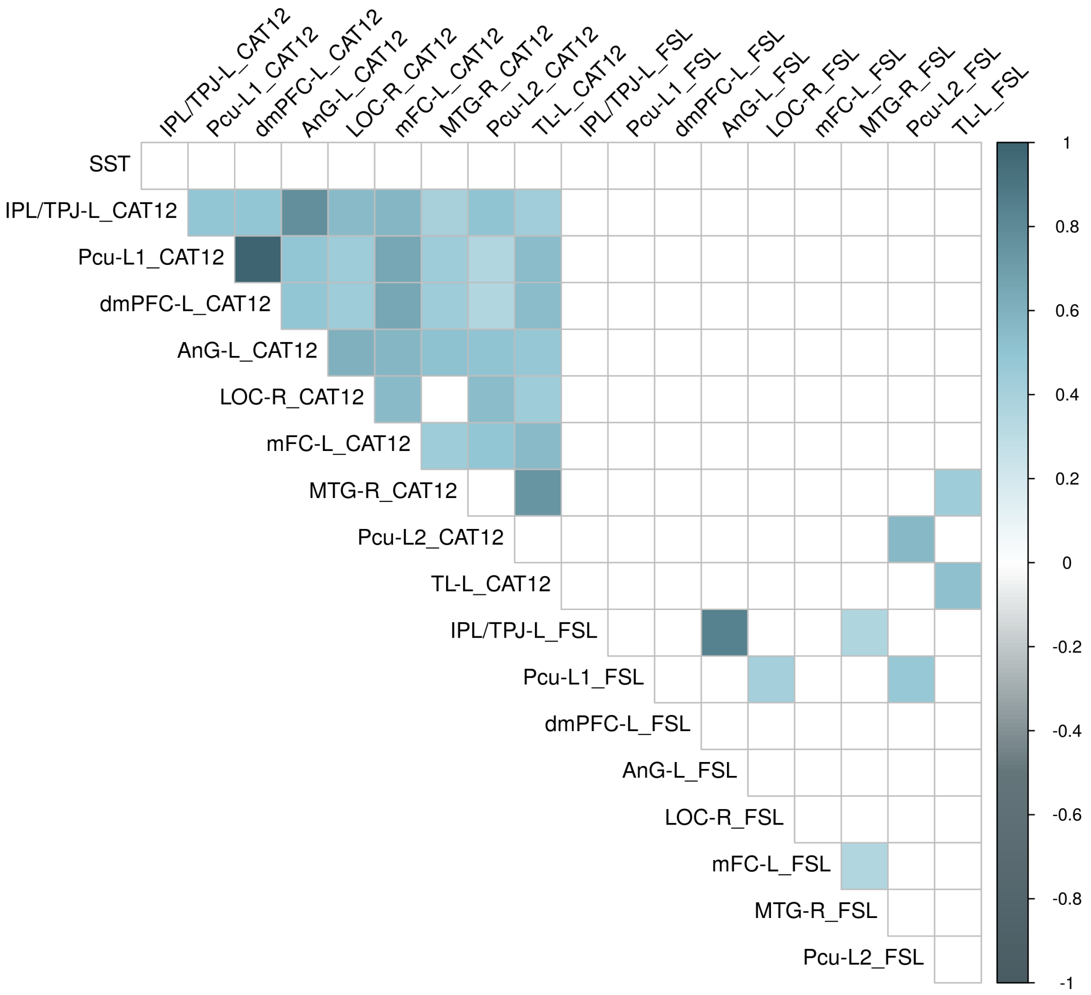

Figure S2. Correlations between extracted volumes (mm^3) of ROI using both softwares (FSL and CAT12), and Theory of Mind scores (SST-MSR) for the full sample (91 participants). Blank spaces mean non-significant results ($p > 0.001$). Abbreviations as in Supplementary Figure 1.

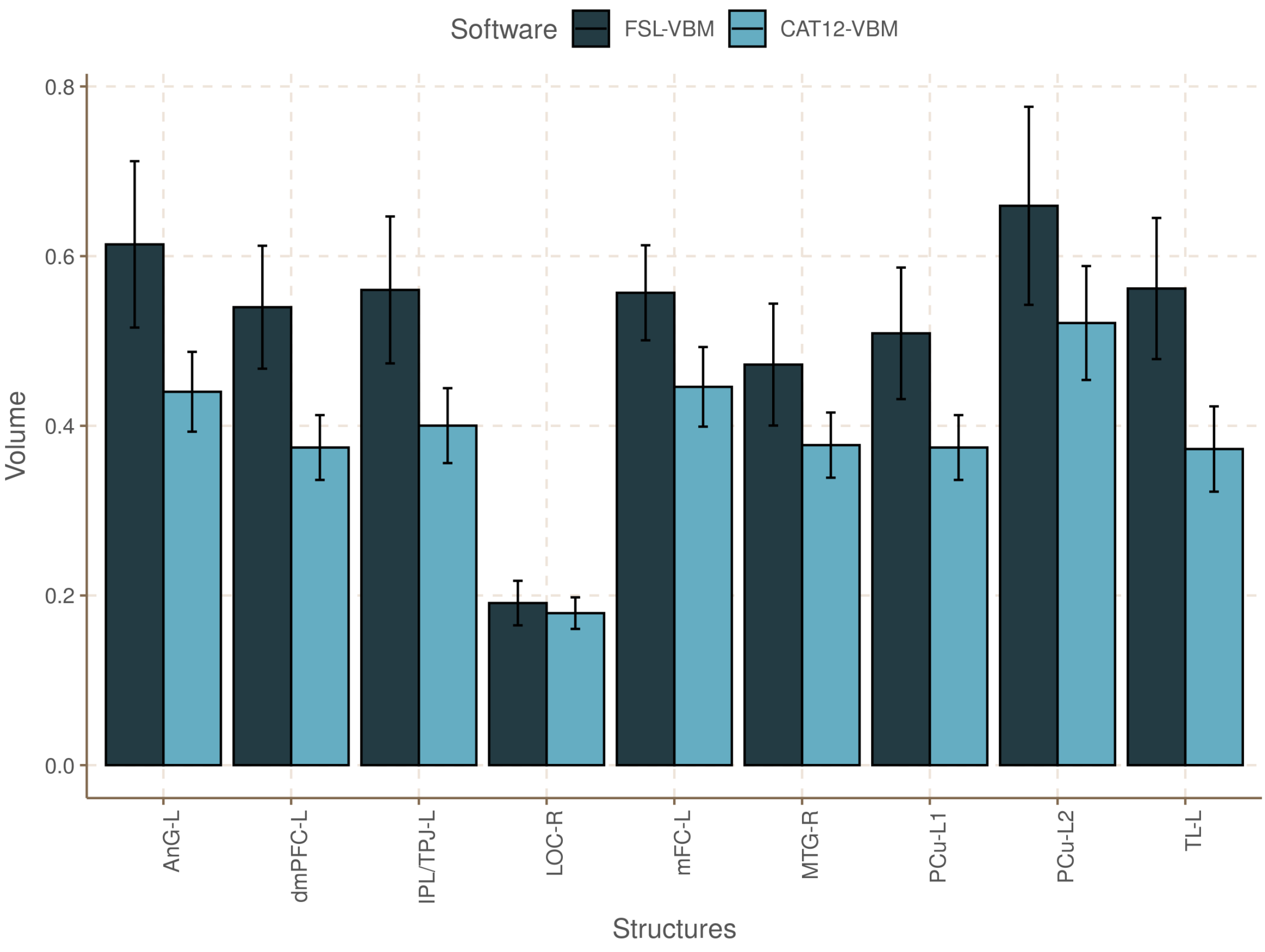

Figure S3. Mean extracted volume values (mm^3) for regions of interest (ROI) for each software. ROI were selected from Neurosynth (Yarkoni et al., 2011) and Sato et al. (2016). Values were significantly higher for FSL for all ROI taken together ($F(8, 1) = 53.25, effectsize(ges) = 0.266, p < 0.001$). Abbreviations as in Supplementary Figure 1.

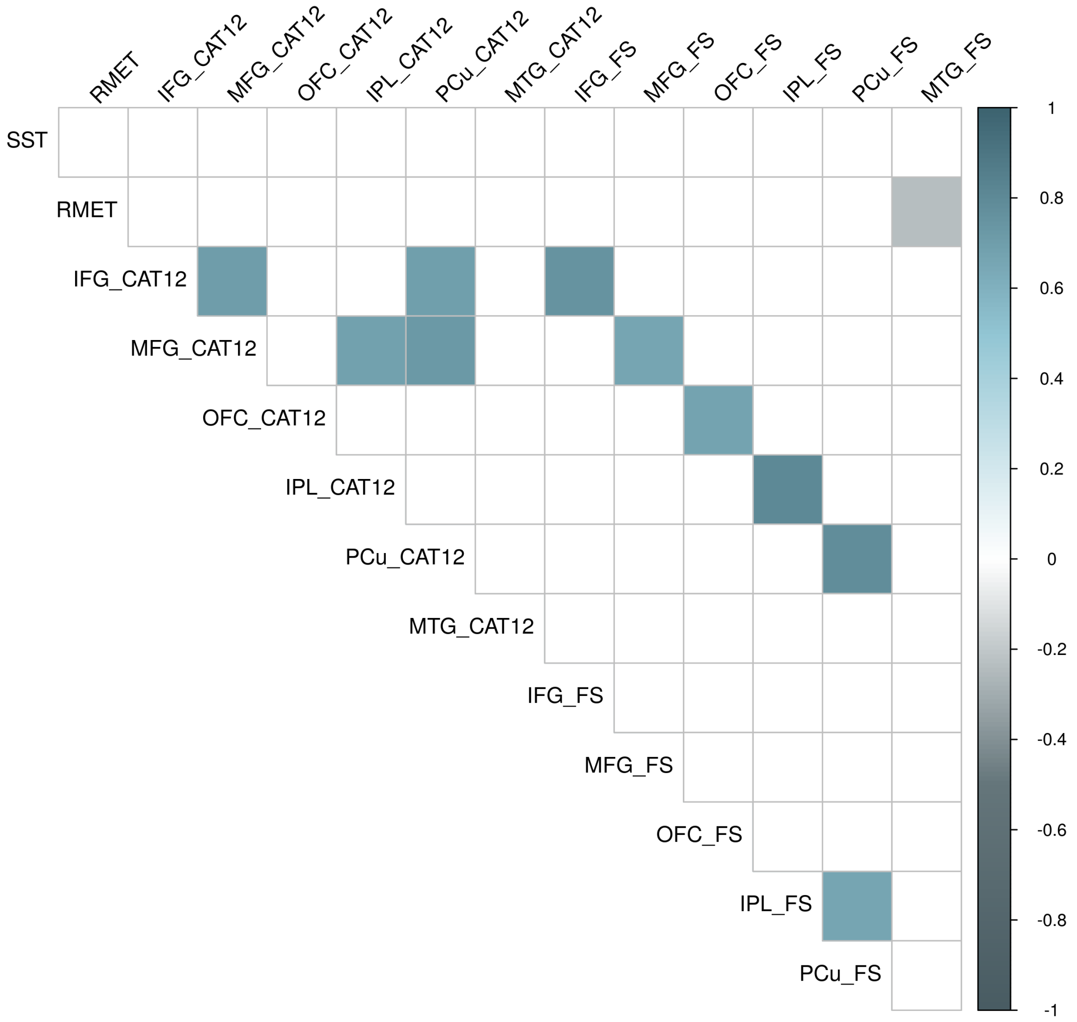

Figure S4. Correlations between extracted cortical thickness (CT) of ROI (mean of both hemispheres), using both softwares (FreeSurfer and CAT12-SBM), and Theory of Mind scores (RMET, and SST-MSR) for the subsample (69 participants). Six of 74 regions of Destrieux atlas were selected considering their association with ToM. Blank spaces means non-significant results ($p > 0.001$). Abbreviations: IFG: Inferior Frontal Gyrus, MFG: Middle Frontal Gyrus, OFC: Orbitofrontal cortex, IPL: Inferior Parietal Lobe, PCu: Precuneus, MTG: Middle Temporal Gyrus, CAT12: Computational Anatomy Toolbox, FS: FreeSurfer.

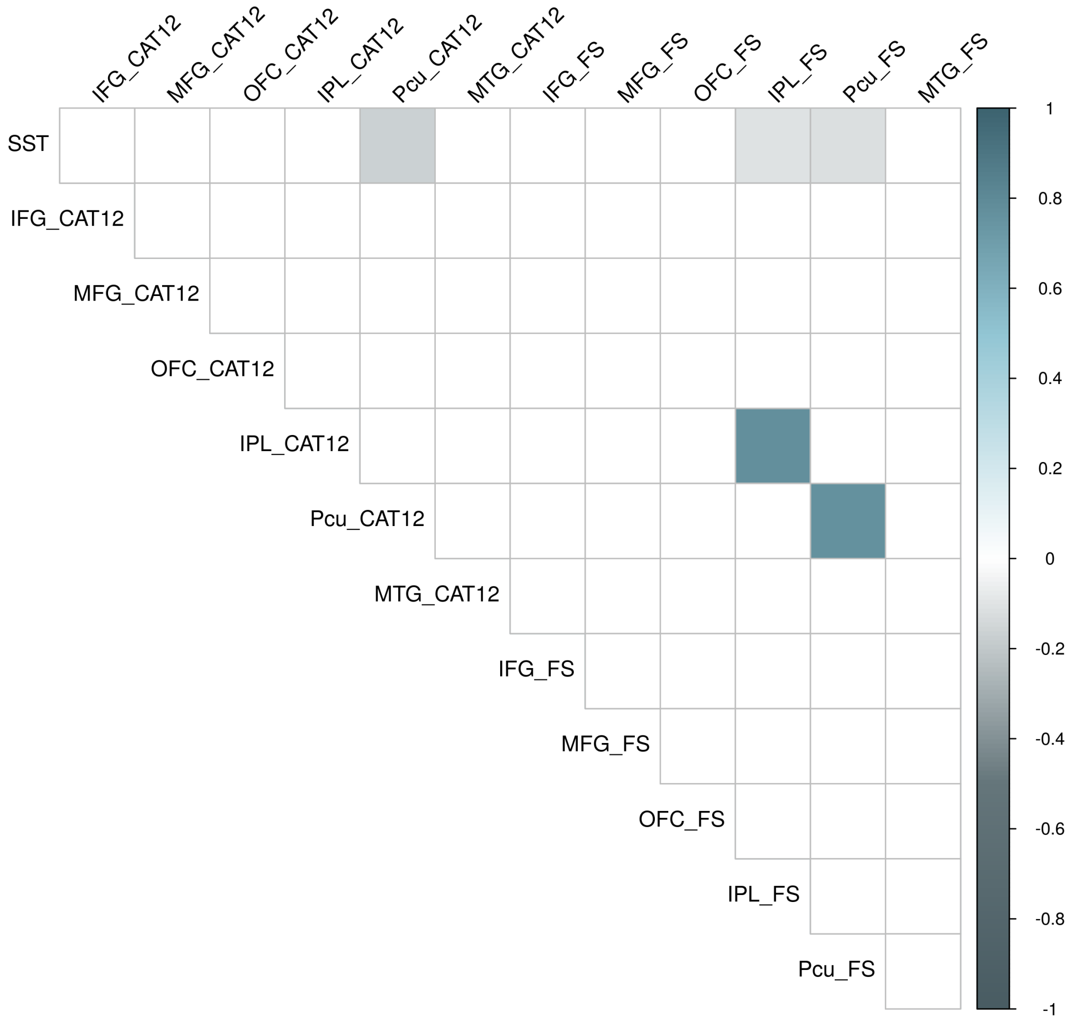

Figure S5. Correlations between extracted cortical thickness (CT) of ROI (mean of both hemispheres), using both softwares (FreeSurfer and CAT12-SBM), and Theory of Mind scores (SST-MSR) for the whole sample (91 participants). Six of 74 regions of aparc.a2009s atlas (Destrieux et al., 2010) were selected considering their association with ToM. Blank spaces means non-significant results ($p > 0.001$). Abbreviations as in Supplementary Figure 4.

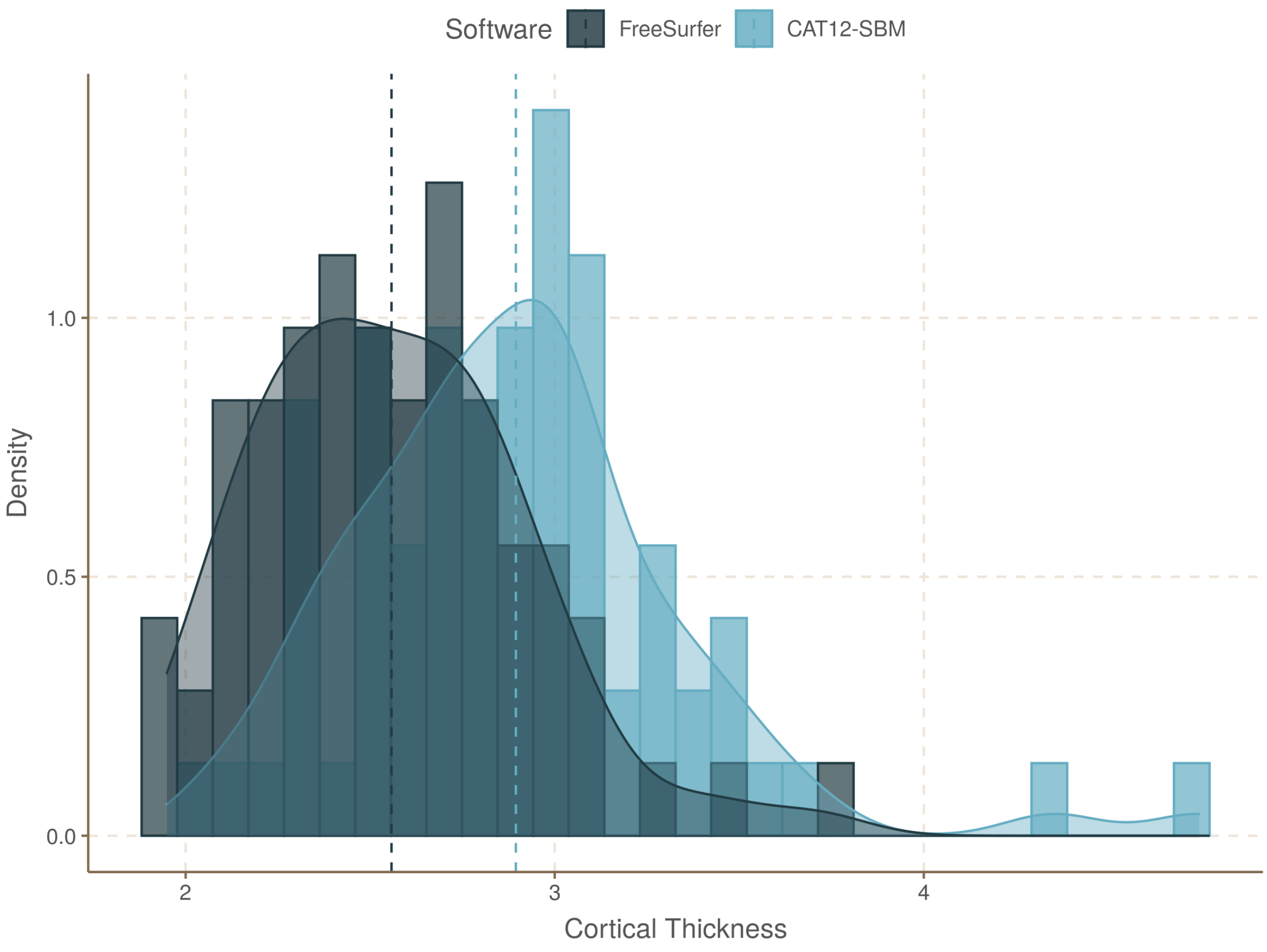

Figure S6. Density distributions of extracted cortical thickness (CT) values (mean of both hemispheres) for each software for 74 regions according to the aparc.a2009s atlas (Destrieux et al., 2010). Dashed lines represent mean values (in mm).

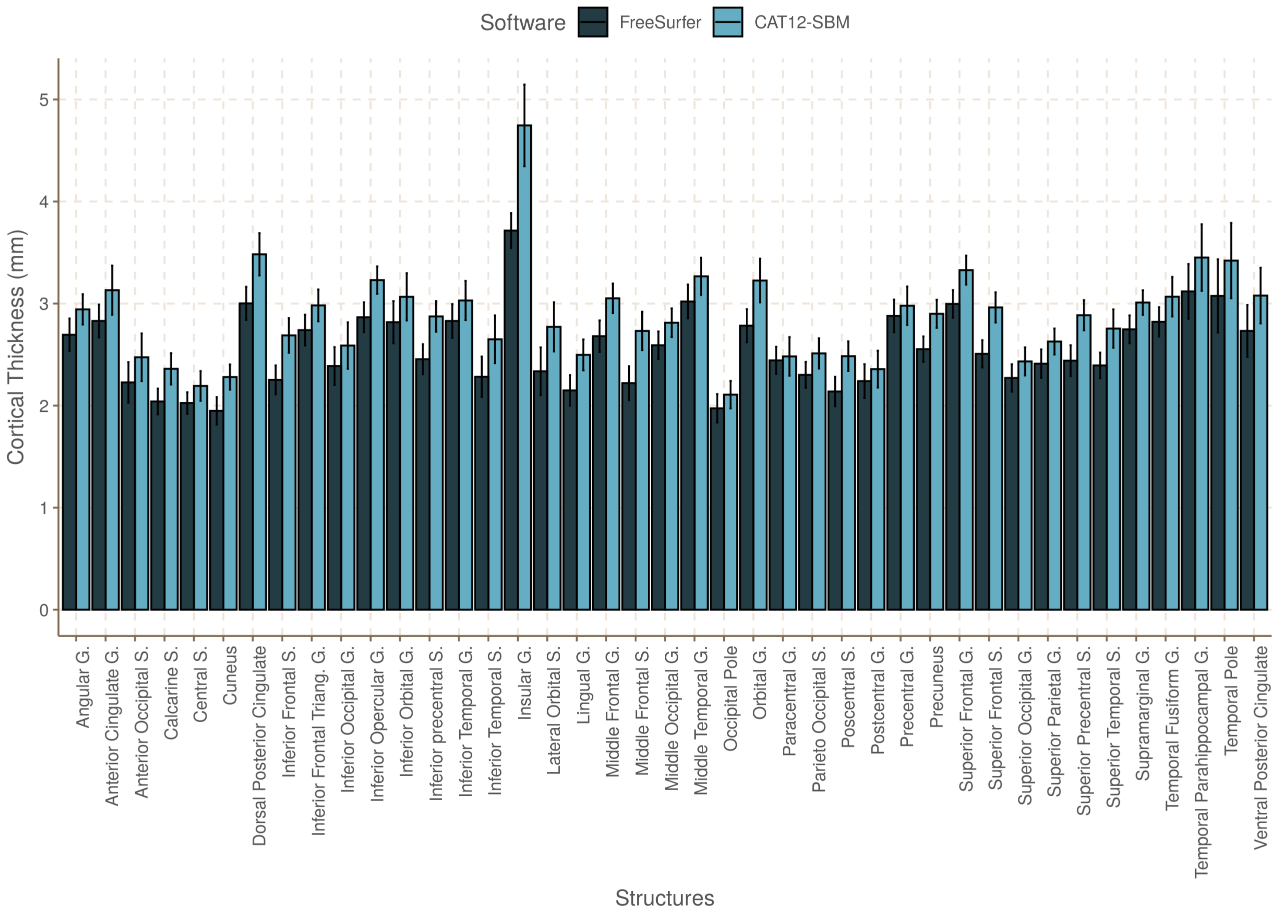

Figure S7. Mean of extracted cortical thickness (CT) values (mean of both hemispheres) using both softwares (FreeSurfer and CAT12-SBM) for 41 of 74 regions according to the aparc.a2009s atlas (Destrieux et al., 2010). These regions were selected only for illustration purposes. Comparisons between FreeSurfer (mean = 2.545 mm) and CAT12-SBM (mean = 2.891 mm) showed higher values for CAT12-SBM ($F(73,1) = 242.3031, effectsize(ges) = 0.155, p < 0.001$).

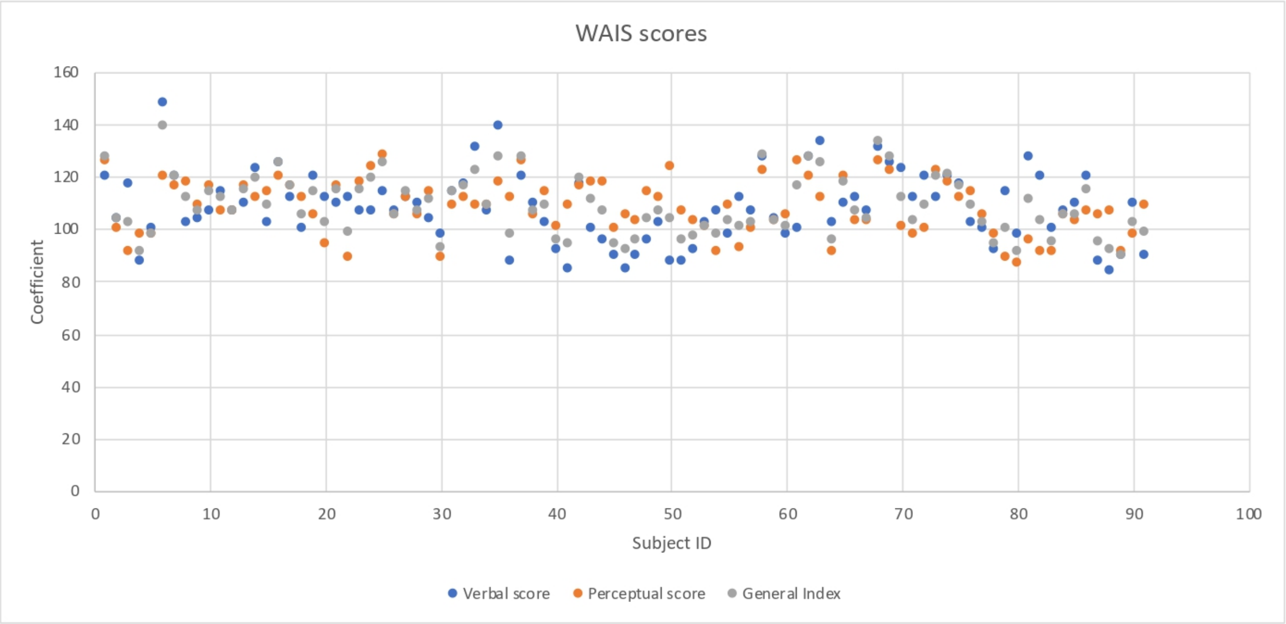

Figure S8. Score distribution of the verbal, perceptual, and general ability indexes of the WAIS test used to characterize the sample. The raw data did not show clusters of participants with higher or lower scores, they fluctuated around the mean, and all were within the neurotypical ranges.
